# Isolation of microglia from retinas of chronic ocular hypertensive rats

**DOI:** 10.1515/biol-2021-0100

**Published:** 2021-09-15

**Authors:** Huimin Zhong, Huan Yu, Jun Sun, Junjue Chen, Shouyue Huang, Ping Huang, Xiaohong Liu, Yisheng Zhong

**Affiliations:** Department of Ophthalmology, Ruijin Hospital Affiliated Medical School, Shanghai Jiaotong University, 197 Ruijin Er Road, 200025, Shanghai, China; Department of Orthopaedics, Shanghai Key Laboratory for Prevention and Treatment of Bone and Joint Diseases, Shanghai Institute of Traumatology and Orthopaedics, Ruijin Hospital, Shanghai Jiao Tong University School of Medicine, 197 Ruijin 2nd Road, Shanghai 200025, People’s Republic of China; Department of Ophthalmology, Shanghai General Hospital (Shanghai First People s Hospital), National Clinical Research Center for Eye Diseases, Shanghai Key Laboratory of Ocular Fundus Diseases, Shanghai Engineering Center for Visual Science and Photomedicine, Shanghai Engineering Center for Precise Diagnosis and Treatment of Eye Diseases, Shanghai, China

**Keywords:** retinal microglia, primary cells separation, COH, Percoll gradient

## Abstract

Microglia are the principal glial cells involved in the processes of immune inflammation within both retina and optic nerve, especially under the context of glaucomatous neuropathy. Considering the distinguishing role of retinal microglia in glaucoma and the lack of established protocol for microglia isolation from animal glaucoma model, the present study aimed to develop and validate a method with characteristics of both simplicity and efficiency for retinal microglia isolation from chronic ocular hypertensive (COH) rats. A Percoll gradient of various concentrations was used to separate microglia from whole retinal cells of the COH rats and control group. The finally isolated microglia were identified by CD11b and Iba-1 immunofluorescence staining, and the cell viability was determined by trypan blue staining. Additionally, the proportion of microglia in the whole retina cells was identified by flow cytometry. Results showed that the survival rates of isolated retinal microglia with the Percoll gradient method were 67.2 ± 4% and 67.6 ± 3% in control and COH groups, respectively. The proportion of the microglia population in the whole retinal cells was about 0.4–0.93%. To conclude, the present study confirmed that the application of Percoll gradient could effectively separate microglia from retinas of COH rats, which will probably enrich the tool kit for basic researchers of glaucoma specialty and help with scientific investigations.

## Graphical abstract

The application of Percoll gradient method could effectively separate microglia cells from retinas of COH rats.

**Figure:**
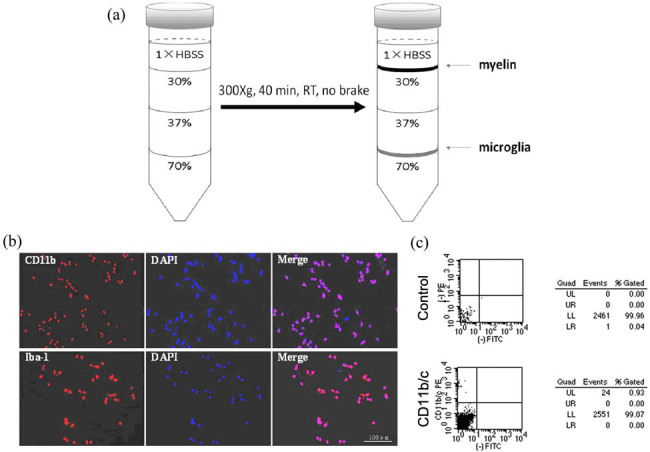

## 1 Introduction

Microglia are macrophages located in the brain and spinal cord as well as in the retina and therein act as innate immune cells. Once the internal environment changes under certain conditions, such as infection and injury, or when subjected to specific physiological or pathological stimuli, the transformation of the microglia in terms of morphology and function will respond immediately [1–5]. It is widely accepted that microglia polarization can be simplified and categorized into the M1 and M2 phenotypes [6]. M1 phenotypic cells usually refer to the microglia, which particularly express pro-inflammatory cytokines (iNOS, TNF-α, IL-1, IL-6, etc.) if stimulated by interferon-γ or TNF-α [7,8]. These cells expand the damage and suppress the repair of tissues within the central nervous system by producing destructive pro-inflammatory mediators. By contrast, M2 phenotypic cells refer to the microglia that express anti-inflammatory cytokines (IL-10, IL-4, etc.) under stimulation of interferon-γ or TNF-α, thus reduce the inflammatory activity and promote the repair of tissues [7].

It is difficult to distinguish microglia from other relevant cell types due to the complexity of identifying and validating the very cellular characteristics, both morphologically and functionally. The morphology and function of microglia are readily impacted by the contents of the surrounding environment, such as neurotransmitters, endocrine factors, specific neurons/astrocytes, etc. [9–12]. In the nervous system, a variety of pathological stimuli (such as ischemia, abnormal protein deposition, infection, etc.) can cause an aggregative response from the microglia, astrocytes, and oligodendrocytes, when inflammatory mediators are released interactively by these glial cells, leading to the nerve inflammation [13–16]. In retinal pathology, microglia are involved in inflammatory processes of various pathological scenarios in the posterior segment [1,2]. How does the transformation of microglia shift between the pro-inflammatory (M1) direction and the anti-inflammatory (M2) direction? Is the increased expression of retinal inflammatory factors principally attributed to the activation of microglia? A series of specific studies focusing on microglia is required to address these issues, and the development of a practical methodology to isolate primary microglia for independent research work becomes necessary.

The isolation and purification of microglia from original tissue are complex. Difficulties encountered in microglia isolation include the relatively small amount of microglia present in tissues, contamination with macrophages, and absence of specific markers differentiating microglia from other blood-derived mononuclear cells [17,18]. There are plenty of protocols described in the literature for the isolation of microglia from the murine brain, canine spinal cord, and canine brain. The effectiveness of the cell sorting procedure substantially depends on the number of viable cells it can process [19–24]. Retinal microglia have also been isolated and purified in rats, dogs, and humans using Percoll density gradient centrifugation and flow cytometry analysis. Dick et al. only used flow cytometry to identify the antigen-presenting cells of normal rat microglia, and some other research subjects were non-pathological dogs [25–27]. However, this experimental tool has not yet been applied to retinas of the glaucoma model, such as rats of induced chronic ocular hypertension. Considering the acknowledged essential role of microglia in the process of glaucomatous neuropathy, and with the aim to facilitate characterizing microglia immunophenotypes and function in the context of glaucoma as well as other relevant retinal diseases, we attempted to isolate the microglia cells from COH rat retinas using the protocol of Percoll concentration gradient centrifugation.

In the present study, the Percoll concentration gradient method was applied to isolate retinal microglia in the COH rat model and provide a new and effective method to facilitate the study of retinal microglia in glaucoma as well as other ocular diseases.

## 2 Materials and methods

### 2.1 Animals

A total of 54 adult male Sprague-Dawley (SD) rats (Slaccas, Shanghai, China) between 200 and 250 g in body weight were used. Rats were housed in a standard animal room with food and water provided ad libitum and a constant room temperature of 22°C (a 12 h light/dark cycle).

**Ethical approval:** The research related to animal use has been complied with all the relevant national regulations and institutional policies for the care and use of animals and was approved by the institutional review board of Ruijin Hospital, Shanghai, China. All animal experiments were carried out in accordance with the ARVO Statement for the Use of Animals in Ophthalmic and Vision Research.

### 2.2 Rat COH model

The COH model was induced in rats as described in our previous work [28]. Briefly, rats were anesthetized with an intraperitoneal injection of ketamine hydrochloride (25 mg/kg; Sigma-Aldrich Corp., St. Louis, MO, USA) and xylazine (10 mg/kg; Sigma-Aldrich Corp.) plus topical administration of 0.5% proparacaine hydrochloride eyedrops (Bausch & Lomb, Tampa, FL, USA). Two dorsal episcleral veins and one ventral episcleral vein in the right eyes of rats were isolated from the surrounding tissues. Each vein was ligated with 10-0 nylon suture (Alcon Laboratories, Ft. Worth, TX, USA) and then severed without damaging the neighboring tissues. The left eyes of these animals were not used as control eyes. An additional group of animals with sham operation served as the control group instead.

Intraocular pressure (IOP) measurements were made under brief systemic anesthesia with isoflurane inhalation (2–4%; Sigma-Aldrich Corp., St. Louis, MO, USA) to minimize the variation caused by stress and movement. IOP was measured in preoperation and postoperation immediately and weekly until the end of the experimental period. To avoid the effect of circadian rhythm, IOP was measured between 10 AM and 2 PM. Each IOP data point was an average value of six consecutive measurements performed with a TonoLab Rebound Tonometer (Icare, Espoo, Finland). We followed the measurement method and conditions described by Morrison et al. [29]. The right eyes with IOP elevation at least 1.3-fold above the baseline during the observation period were considered as the COH group. Considering the failure of the model and other factors, the right eyes whose IOP increased less than 1.3 times have been excluded. IOP of the left eyes remained at approximate baseline.

### 2.3 Retina collection

Four weeks after the induction of COH, the COH model rats and the control rats were anesthetized with an intraperitoneal injection of ketamine hydrochloride (25 mg/kg; Sigma-Aldrich Corp.) and xylazine (10 mg/kg; Sigma-Aldrich Corp.) plus topical administration of 0.5% proparacaine hydrochloride eye drops (Bausch & Lomb). A portion of the skin on the neck side of the rat was cut, exposing the jugular vein. Then the left chest skin and the ribs were also cut and broken to open the chest. A three-way tube was inserted through the left ventricle into the ascending aorta. The right atrial appendage was cut open for drainage. Meanwhile, precooled 0.9% normal saline was perfused through the three-way tube for removing the mononuclear cells from the retinal circulation until the jugular vein changed from purple to white. After perfusion for several minutes, the right eyeball was taken out by using tweezers to lift the conjunctiva from the optic nerve and placed in the ice-cold sterile Hanks’ balanced salt solution (HBSS) solution (Invitrogen, USA) containing 1% streptomycin (Invitrogen, USA). The eyeball was washed with HBSS three times, then was cut around 1–2 mm away from the corneal limbus with sterile microsurgical scissors and microscopic tweezers. The cornea was removed by making a circle around the eyeball along the incision, and then the lens, ciliary body, and vitreous were gently separated in sterile phosphate-buffered saline (PBS). Finally, the retina could be isolated from the optic cup and placed in a 15 mL-size tube with sterile HBSS solution.

### 2.4 Retina digestion

Collagenase/Dispase (10269638001, Roche, Germany) was dissolved in sterilized double-distilled water to prepare a stock solution at a 100 mg/mL concentration, stored at −80°C after dispensing. The stock solution was diluted to 1 mg/mL concentration with sterilized double-distilled water. Then, Dnase I (Sigma) was added to a final concentration of 50 u/mL for use. After the tube containing retinas was treated with fixed angle centrifugation at a low speed of 1,000 rpm (300×*g*), the supernatant was removed, then 1 mL digestive enzyme was added to the tube. The retinas were digested for 30 min at 37°C and were shaken gently several times every 5 min via inversion of the tube. After completion of the digestion, the centrifuge tube was cooled in ice and centrifuged 5 min at 300×*g*. The supernatant was discarded, then HBSS (18°C) was added and centrifugated for 5 min at 300×*g*. The previous step was repeated, and the pellet was then resuspended in 4 mL 37% Percoll (GE Healthcare, USA) at room temperature.

### 2.5 Preparation of Percoll solution with different concentrations (densities)

1× HBSS/10 mM HEPES [4-(2-hydroxyerhyl)piperazine-1-erhaesulfonic acid] solution preparation: 5 mL of 1 M HEPES (Invitrogen, USA) was added into 495 mL of 1× HBSS and mixed upside-down at room temperature. Stock isotonic Percoll (SIP) solution preparation: 18 mL of stock Percoll solution (GE Healthcare, USA) was added into 2 mL of 10× HBSS and mixed upside-down at room temperature, and the SIP solution was defined as 100% Percoll solution. Seventy percent (70%) Percoll solution preparation: 7 mL of SIP solution was added into 3 mL of 1× HBSS/10 mM HEPES and mixed upside-down at room temperature. Thirty-seven percent (37%) Percoll solution preparation: 3.7 mL of SIP solution was added into 6.3 mL of 1× HBSS/10 mM HEPES and mixed upside-down at room temperature. Thirty percent (30%) Percoll solution preparation: 3 mL of SIP solution was added into 7 mL of 1× HBSS/10 mM HEPES and mixed upside-down at room temperature [30,31].

### 2.6 Sample loading

**Figure 1:**
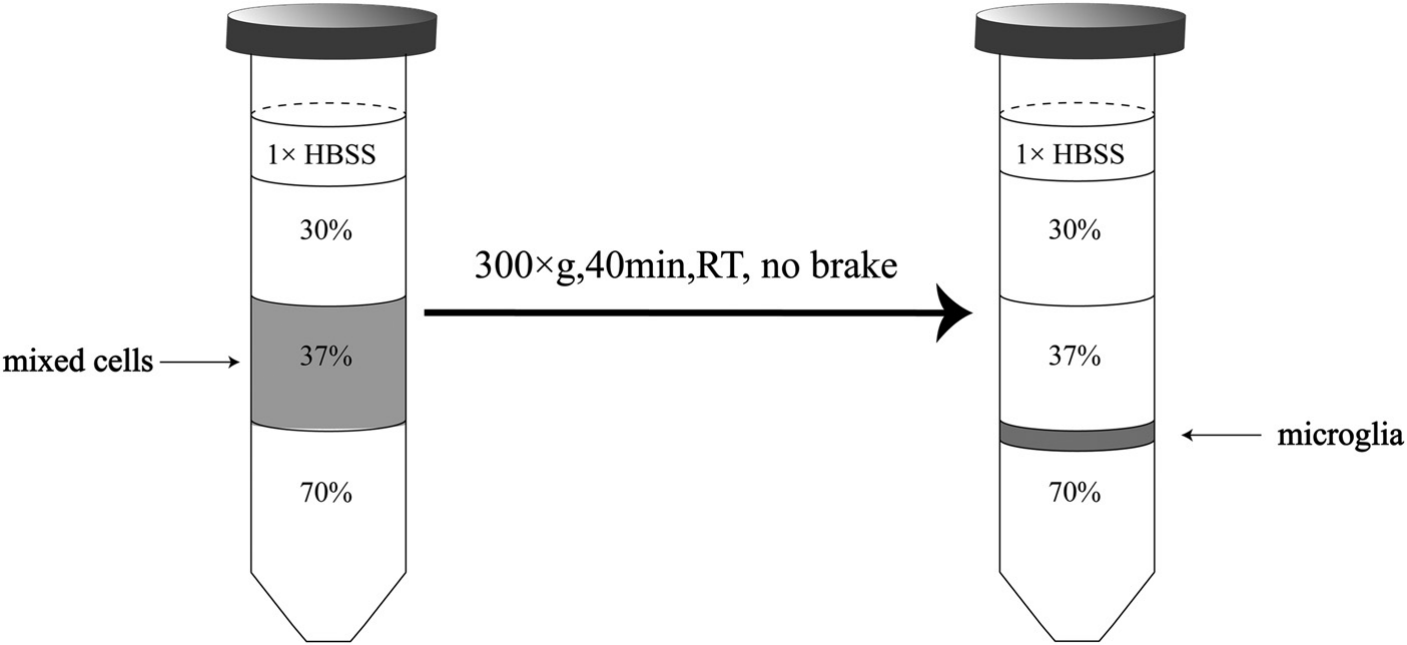
The schematic diagram of different concentrations of (density) Percoll solution in 15 mL centrifuge tube.

First, 4 mL of 37% Percoll solution suspended with mixed cells was added to the bottom of the 15 mL centrifuge tube (cell layer). Second, 4 mL of 70% Percoll solution was gently added under the cell layer using a pipette. Third, 4 mL of 30% Percoll solution was slightly added above the cell layer. Finally, 2 mL of 1× HBSS was added above 30% Percoll solution (Figure 1).

### 2.7 Centrifugation and sampling

The tube with loading sample was centrifuged for 40 min at 300×*g* (18°C). Make sure the centrifugation was carefully stopped with no brake to avoid interrupting the interphase. The majority of the cells to be separated were located in the interface liquid between the 70% Percoll solution layer and 37% Percoll solution layer. The interface liquid (about 2 mL in volume) was collected after gently removing the Percoll solution above the interface, was immediately added to 6 mL of precooling HBSS, and was further centrifuged for 20 min at 300×*g* (18°C) to get rid of the Percoll solution. After removing the supernatant, the pellet was resuspended in 1 mL of HBSS for cell counting.

### 2.8 Cells count

A clean coverslip was gently mounted on the counting chamber of the hemocytometer. A proper amount of cell suspension was transferred into the counting chamber along the lower margin of the coverslip using a pipette. The cells were observed with microscope 1–2 min after the transfer when the cells settled down and no longer drifted with the liquid. The hemocytometer was first observed under the microscope at low magnification to locate the counting area, and then the magnification was switched to high level for further observation and counting. In order to avoid miscounting and recounting, the cells located on the upper and left lines of the grid were deemed to be included in the grid, while the cells located on the below and right lines of the grid were deemed to be not included in the very grid but left for the neighboring grid.

\text{Cell}\hspace{.5em}\text{concentration}\hspace{.5em}(\text{cells}/\text{mL})=\text{Total}\hspace{.5em}\text{number}\hspace{.5em}\text{of}\hspace{.5em}\text{four}\hspace{.5em}\text{large}\hspace{.5em}\text{grids}/4\times {10}^{4}\times \text{Dilution}\hspace{.5em}\text{multiples}\text{.}]

### 2.9 Determination of cell viability

The cells separated from the retina of the COH rat with the Percoll gradient concentration method were resuspended in 1 mL of HBSS. Then 100 μL of cell suspension was transferred into a 1.5 mL centrifuge tube, followed by the addition of 100 μL of 2× trypan blue solution (Beyotime, Shanghai, China). The blended liquid was gently mixed and stained for 3 min before being transferred into the counting chamber, and the total number of cells and the number of blue-stained dead cells were counted under a microscope. Cell viability = (total number of cells − number of blue-stained cells)/total number of cells × 100%. Cell viability assessment was done before cell platting for every round of microglia culturing, and cells were viable at every stage of this protocol.

### 2.10 Identification of microglia using immunofluorescence

The isolated glial cells composed of microglia mostly, along with a small proportion of Müller cells and astrocytes, were planted in the 24-well plate covered with polylysine-coated glass slides after the cell concentration was adjusted to 1 × 10^5^–10^7^/mL. After being cultured for 24–48 h, most of the cells were attached. The cells were washed with PBS three times, then fixed with 4% paraformaldehyde at room temperature for 30 min. After washing with PBS, the blocking solution (containing 5% donkey serum and 0.1% Triton X-100 PBS) was added to the cells and incubated at room temperature for 2 h, and then the blocking solution was discarded. The cells were subsequently incubated with 100 μL of goat anti-rat Iba-1 polyclonal antibody (1:500, Abcam USA) or rabbit anti-rat CD11b polyclonal antibody (1:250, Abcam) overnight at 4°C. The cells were washed with PBS three times (5 min per time), then incubated with FITC-conjugated donkey anti-goat (1:100, Invitrogen) or Cy3-coupled donkey anti-rabbit (1:100, Biolegend, USA), respectively, according to the selection of primary antibody, at room temperature for 1 h. Following three washes (5 min per time) of PBS, the coverslips were mounted on the glass slides with Histomount TM (Invitrogen). Finally, the cells were observed under a fluorescence microscope, and images were acquired with a digital camera (magnification 200×, Carl Zeiss Microscopy).

### 2.11 Flow cytometry to identify the proportion of microglia in the retina

Retinas were collected and digested in the same way as the steps previously mentioned. Immediately after the termination of digestion, the centrifuge tube containing the digested retina was cooled in ice and then centrifuged at 500×*g* at 4°C for 5 min. After the supernatant was discarded, the pellet was resuspended in 500 μL of precooled HBSS. Subsequently, the resuspended cell suspension was centrifuged at 800×*g* at 4°C for 5 min; and repeated step 1 time. After the supernatant was discarded again, the pellet was resuspended in 100 μL of flow buffer (eBioscience, USA). The classification of gating was based on Isotype Control which was equivalent to the negative control of the experiment. Thus, we chose mouse IgG2A for gating. The flow antibody anti-rat CD11b/c (0.125 μg/tube, Mouse IgG2A, eBioscience) was added to the sample tube; the flow antibody mouse IgG2A (0.125 μg/tube, eBioscience, USA) was added to the control tube. After mixing gently, the tubes were placed in the dark at 4°C for 30 min, and then centrifuged at 4°C and 300×*g* for 5 min. After the supernatant was discarded, the stained cell pellet was resuspended in 100 μL of flow buffer and centrifuged at 4°C and 300×*g* for 5 min, and this step was repeated once more. The stained cell pellet was resuspended in flow buffer, and then placed on ice before analyzed with flow cytometry.

### 2.12 Statistical analysis

All data are expressed as the arithmetic mean ± SD. Statistical analyses were performed by using SPSS software (IBM SPSS statistics Version 19.0; IBM, Armonk, NY). The two-tailed independent-samples test was used to compare the IOP, the numbers of microglia yield, and the cell survival rate between the COH and control groups. *P* < 0.05 was regarded as statistically significant.

## 3 Results

### 3.1 The animal usage and profile of IOP measurement

A total of 54 rats were subjected to operations throughout this study. However, eight rats that failed to meet the qualifying criterion for COH along with two rats that suffered severe inflammation or ocular infection were excluded. Ultimately, 44 rats were used for the study, and the number of animals used in various procedures is specified in Table 1. The arithmetical mean IOP of each group at the various time points was listed in Table 2.

**Table 1:** Number of animals used in separate procedures

Procedure	COH group (*n*)	Control group (*n*)
Cells count	9	6
Cells identification	5	6
Determination of cells viability	6	6
Flow cytometry	3	3

**Table 2:** Summary of IOP measurements in grouped rats (Mean ± SD)

Time	COH group (*n* = 20)	Control group (*n* = 18)	*P*
Week 1	27.8 ± 3.1	13.8 ± 1.6	<0.001
Week 2	25.8 ± 2.4	14.2 ± 1.5	<0.001
Week 3	24.7 ± 2.1	13.7 ± 1.4	<0.001
Week 4	22.4 ± 1.8	14.0 ± 1.7	<0.005

Unit: mm Hg.

### 3.2 The numbers of microglia isolated from COH model rat retina

The amount of primary retinal microglia isolated from the COH rat and the control group using the Percoll gradient concentration method was relatively limited. The number of microglia isolated from the control group was (83,334 ± 10,801) cells/retina. Four weeks following the induction of COH, the number of microglia isolated from the COH tgroup was (106,842 ± 9,698) cells/retina, which was significantly higher than that of the control group (*t* = −3.967, *P* = 0.003).

### 3.3 Identification of isolated microglia with immunofluorescence

**Figure 2:**
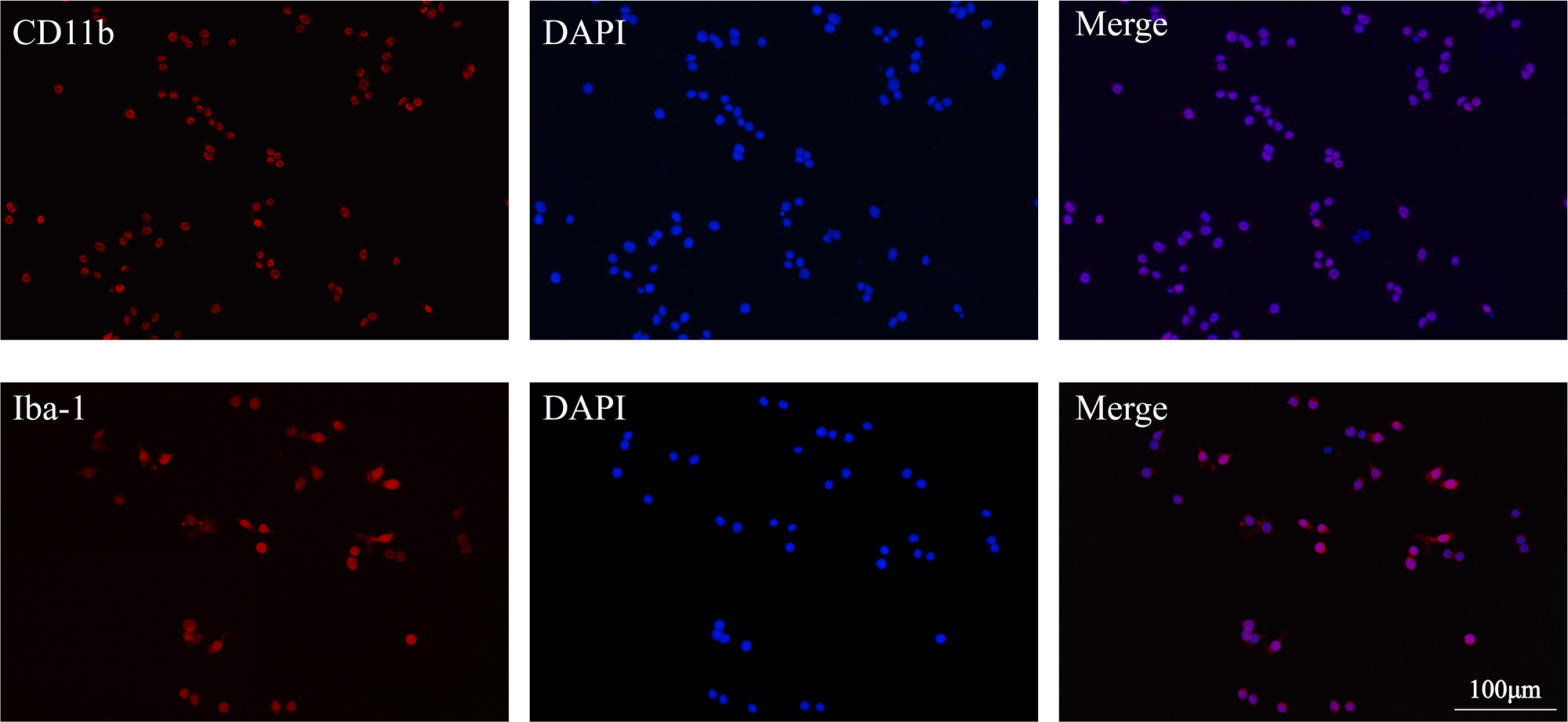
Identification of isolated microglia through CD11b and Iba-1 immunofluorescence (magnification 200×, scale bar = 100 μm).

The recognition of the isolated cells as microglia was validated with immunofluorescence staining of CD11b and Iba-1, which were acknowledged characteristic markers for microglia. The result confirmed that the isolated cells were mostly identified as retinal microglia (Figure 2), and the application of the Percoll gradient concentration method presented promising efficacy and simplicity in yielding primary retinal microglia in the experimental glaucoma model.

### 3.4 Cell viability

The cell survival rates of isolated microglia by the Percoll gradient concentration method were 67.2 ± 4% and 67.6 ± 3% in control and COH groups, respectively, and no significant difference was found between the two groups (*t* = −0.244, *P* = 0.812).

### 3.5 Identification by flow cytometry

**Figure 3:**
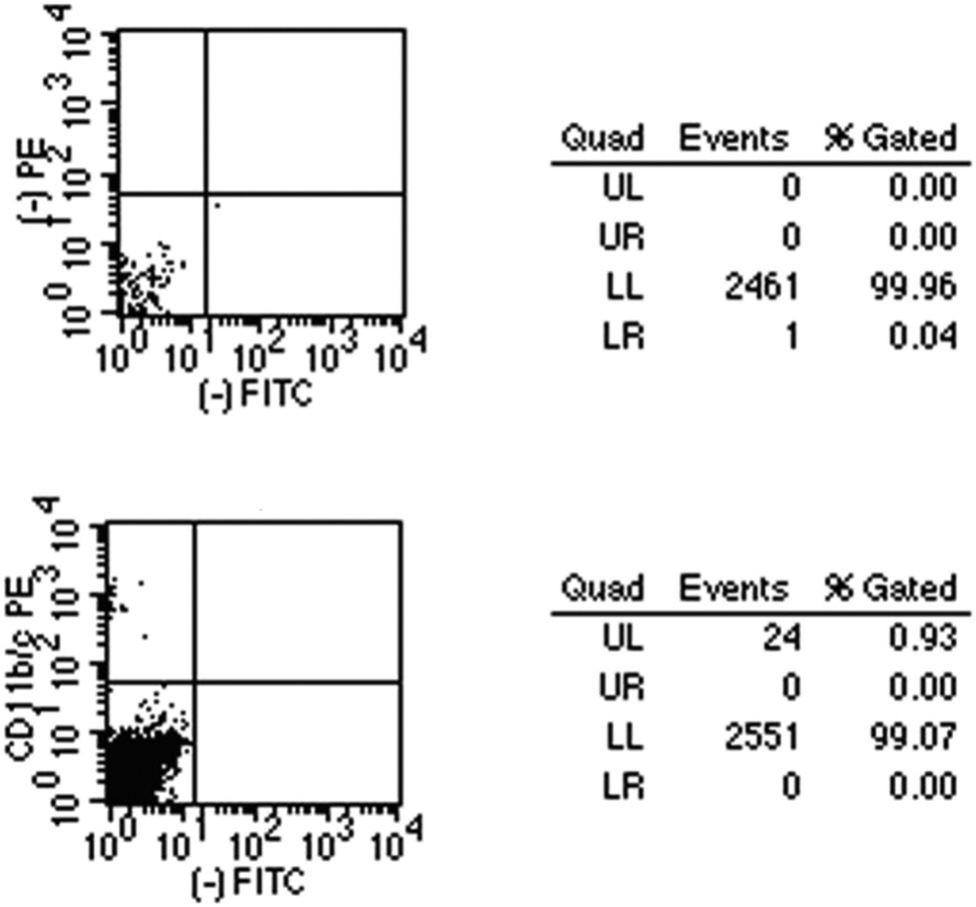
The proportion of microglia in the retina was identified by flow cytometry. The above picture was blank control, the lower picture was the CD11b/c flow antibody staining positive cells. UL: upper left; UR: upper right; LL: lower left; LR: lower right.

The proportion of microglia in the whole retina cells after initial digestion, similarly between the control and COH groups, was about 0.4–0.93% (Figure 3), yet no more positive than 1% was identified as microglia even after repeating three times of the procedure. It would be helpful to have information regarding the proportion of microglia in the COH retina, as it appears that it would be substantially higher. However, the ratio did not change much because the total number of cells also increased.

## 4 Discussion

In this study, we successfully isolated retinal microglia from COH rats with the Percoll gradient concentration method. Given the deep involvement of microglia in the physiopathologic processes within the central nervous system, methods for extracting microglia from the brain have been widely developed and reported [2,30,32,33]. However, there are limitations in these methods, such as expensiveness and complexity of the operation, which unavoidably impair the feasibility of this research tool. Moreover, from the viewpoint of researchers engaged in eye-related basic science, the lack of microglia-isolating protocol specialized for retina may appear to be the most notable inadequacy in this field, since retina is the unique observable tissue in the central nervous system, and this advantage makes adult rat modeling of retinal diseases an excellent reference for the study of the central nervous system in terms of both structure and function. Therefore, our research is devoted to remedy this inadequacy and try to develop a practical methodology as a research tool to facilitate associated scientific work, which focuses on potential mechanisms that microglia may be involved in the posterior segment of the eye, especially in the condition of glaucoma [34,35]. The density of isolated microglia obtained in different tissues is various. For the retina as the primary source of separated microglia, the sample size has to be expanded to meet routine cytological experiments due to the relatively low yield of the target cells. Moreover, we believe that primary cells have meaningful advantages over cell lines; for instance, primary cells are likely to exhibit physiological and pathological behaviors of more similarity to their natural feature.

Percoll is polyvinyl pyrrolidone-coated silica particle with a diameter of 20–22 nm under the electron microscope. Liquid kinetic measurements show that the diameter of Percoll is 29–30 nm in 0.15 M NaCl solution, while 35 nm in water. It presents a low osmotic pressure (<20 mosm/kg H_2_O), a small viscosity, and a high density of up to 1.3 g/mL. With the application of Percoll gradient, the distinctive cells can be well-separated at a low centrifugal force (200× to 1,000×*g*) for several minutes. Its low diffusion constant contributes to a stable state of Percoll gradient. In addition, owing to its inability to penetrate biofilm and nontoxicity to cells, Percoll is widely used for the separation of cells, subcellular components, bacteria, and viruses. Percoll can also be utilized to separate damaged cells and cellular fragments from intact living cells. However, Percoll would not be able to separate cells properly below normal temperature due to structural transformation of clumping. Compared with flow separation, Percoll gradient concentration method is more economical and highlighted in the advantage of avoiding immune response caused by antigen-antibody binding.

Our research shows that the amount of microglia isolated from normal rat retina by Percoll gradient concentration was limited; an average of (83,334 ± 10,801) cells/retina yield each time. The amount of microglia isolated from COH rat retina by Percoll gradient concentration was significantly more than that from normal ones; however, it was also limited, an average of (106,842 ± 9,698) cells/retina yield each time. It has been widely acknowledged that microglia are deeply involved in the pathological process, IOP-related glaucomatous optic neuropathy. Neufeld found that microglia were activated in the optic disc head and the parapapillary region with altered morphology and distribution in condition of glaucoma [36]. Wang et al. found that activated microglia appeared in the ganglionic layer of the optic nerve only 2 h after induction of IOP elevation in animal glaucoma model, and the number of cells increased with time [37]. Naskar et al. observed that microglia engulfed fluorescently labeled retinal ganglion cells in eyes of glaucoma rats, and that the microglia gathered around the surviving retinal ganglion cells [38]. Our recent study also found that microglia were activated in the early stage of COH in rat retina, featured by the increase of cell amount and alteration of morphology from slender branching to spherical amoeboid [28]. Retinal microglia activation in the COH rat can explain why the amount of retinal microglia isolated from COH rat was higher than that from normal ones.

Given the relatively low yield rate of target cells, we conducted a flow cytometry analysis of the whole retina cells, and the majority of the cells digested out of the retina turned to be various types of retinal glial cells, including a large proportion of Müller cells, astrocytes, and a small proportion of microglia. The distinctive microglia and total cell count from the flow assessment were 24 and 2,575, respectively, but only 0.4–0.93% of the cells were found to be microglia. Szabo et al. reported that the percentage of microglia obtained from forebrain of fetal SD rat was less than 0.5% [39], which was similar to our finding in retina. In the study of Dick et al., the retinas were disrupted mechanically through a stainless steel sieve to isolate the microglia, and flow cytometry analysis revealed that the proportion of microglia was up to 8% [25]. We speculate that: (1) In *in vitro* culture, microglia grow semi-adherently, resulting in the loose combination of microglia with extracellular matrix, thus fall off more easily when encountered mechanical extrusion. (2) Enzyme digestion may exert more impact on microglia survival and manifestation of its surface antigens than in the scenario of mechanical extrusion. These two aspects can probably explain why Dick et al. obtained a higher proportion of microglia than Szabo et al. did [25,39].

Whichever method is used to separate microglia, the total yield of microglia in each retina is very low. According to the latest research, only 25% of the cells obtained by Percoll gradient concentration were microglial cells [40], which means the amount of microglia was 1.75 × 10^3^–2.5 × 10^4^ cells/retina. This yield rate will fail to provide enough material for further experiments of molecular biology, such as Western blot, flow cytometry, PCR, etc., when considering the cell loss, and inevitably call for unpractical increase of animal sample size.

There are many methods for the isolation of microglia, including Percoll gradient, fluorescence activated cell sorting (FACS) and magnetic-activated cell-sorting (MACS), etc. [19,20,25–27]. FACS is based on the labeling of cells with fluorescence-tagged biochemical antibodies, which may appear to be a more attractive option when sorting cells intended for further precise applications such as RNA-sequencing [21,22]. However, it is relatively time-consuming when there is the need of very large cell numbers and requires expensive machinery. MACS could purify the cells by using magnetic beads conjugated to specific antibodies, which may be more suitable for time-sensitive experiments since it can only target one or two antigens [41,42]. Percoll gradient for the isolation of microglia is performed in less time-consuming manner and requires less expensive equipment, which is suitable for further flow cytometry analysis, RNA isolation for gene expression by real-time PCR or microarrays, and for functional assays including cytokine production, chemotaxis, and phagocytosis [2]. In this study, Percoll gradient was used to isolate retinal microglia, in similar way as the method presented by Genini et al. [26]. We uniquely apply this method to the isolation of retinal microglia in COH rats.

In summary, the Percoll gradient of various concentrations could be applied to effectively separate microglia from retinas of experimental glaucoma rat indeed, although the proportion of microglia yield in the whole retinal cells was relatively low. The amount of modeling subjects has to be increased to provide enough microglia for subsequent experiments.
